# Supplementary material for: Global trends in smoking-attributable rheumatoid arthritis burden: Insights from GBD 2021
Source: PLoS One. 2025 Aug 5;20(8):e0329434. doi: 10.1371/journal.pone.0329434 (PMC12324138; doi:10.1371/journal.pone.0329434)
Supplement: S1 Data — This compressed ZIP archive contains all underlying data required to reproduce the figures and tables presented in the manuscript. (ZIP) [file pone.0329434.s001.zip › S1_Data/Figure1/ne_110m_admin_0_countries/ne_110m_admin_0_countries.README.html]

Natural Earth » Blog Archive » Admin 0 – Countries - Free vector and raster map data at 1:10m, 1:50m, and 1:110m scales 


# 

Free vector and raster map data at 1:10m, 1:50m, and 1:110m scales

Search for:

- Home
- Features
- Downloads
- Blog
- Issues
- Corrections
- About

---

## Admin 0 – Countries

*There are **258 countries** in the world. Greenland as separate from Denmark. Most users will want this file instead of sovereign states, though some users will want map units instead when needing to distinguish overseas regions of France.*

*Natural Earth shows **de facto** boundaries by default according to who controls the territory, versus *de jure*.*

Download countries (210.08 KB) version 5.1.1

Download without boundary lakes (212.3 KB) version 5.1.1

**About**

Countries distinguish between metropolitan (homeland) and independent and semi-independent portions of sovereign states. If you want to see the dependent overseas regions broken out (like in ISO codes, see France for example), use map units instead.

Each country is coded with a world region that roughly follows the United Nations setup.

Includes some thematic data from the United Nations, U.S. Central Intelligence Agency, and elsewhere.

**Disclaimer**

Natural Earth Vector draws boundaries of countries according to defacto status. We show who actually controls the situation on the ground. Please feel free to mashup our disputed areas (link) theme to match your particular political outlook.

**Known Problems**

None.

**Version History**

- 5.1.1
- 5.1.0
- 5.0.1
- 5.0.0
- 4.1.0
- 4.0.0
- 2.0.0
- 1.4.0
- 1.3.0
- 1.1.0

The master changelog is available on Github »

This entry was posted
on Monday, September 21st, 2009 at 10:21 am and is filed under 110m-cultural-vectors.
You can follow any responses to this entry through the RSS 2.0 feed.
Both comments and pings are currently closed.

- Comments (3)
- Trackbacks

1. Getting rasters into shape from R | John Baumgartner's blog says:

   July 26, 2012 at 11:32 pm

   […] earlier. It’s the result of a conversion of a polygon shapefile of country boundaries (from Natural Earth, a fantastic, public domain, physical/cultural spatial data source) to a raster data […]
2. jQUERY e Mappe: jVectorMap | SimPoli says:

   February 28, 2013 at 5:42 am

   […] Le mappe sono scaricate da https://www.naturalearthdata.com […]
3. SimPoli » jQUERY e Mappe: jVectorMap says:

   March 13, 2013 at 2:52 am

   […] Le mappe sono scaricate da https://www.naturalearthdata.com […]

- Subscribe: Entries | Comments
- ## Search

  Search for:
- ## Links

  - NACIS
- ## Tags

  10m
  50m
  90
  180
  admin-0
  bjorn
  bounding box
  browser
  change log
  corrections
  countries
  Downloads
  error
  extent
  ext js
  forums
  geoext
  hans
  imagery
  import
  mapnik
  maptiler
  map tiles
  marine boundary
  national parks
  new data
  nsd
  openlayers
  physical labels
  pngng
  populated places
  raster
  terrestrial hypsography
  tfw
  thematic mapping
  themese
  tif
  tilecache
  tiles
  time zones
  towns
  transportation
  update
  visitors
  world file
- ## Recent Comments

  - Aligning Natural Earth Geojson and Raster to render in D3 – BBSCODE on 1:50m Shaded Relief
  - QGIS a Mapping Tool – April Dahn on 1:50m Shaded Relief
  - More Mapping with QGIS – History 502 on 1:50m Shaded Relief
  - buy ivermectin 12 mg tablets on Download URLs – double slash
  - Building A Beautiful And Clear Map From Massive, Complex Data – Slacker News on 1:10m Shaded Relief
- ## Recent Forum Topics

  - Natural Earth in Wagner VII
    by Hugo Ahlenius
  - Downloads are 404ing
    by Nathaniel
  - Disputed Territories: "type" field
    by alykat
  - ISO code confusion
    by nth
  - Bad ADM1NAME, encoding in version 3.0.0 and missing diacritics in NAME
    by pfunes
  - U.S. County Shape File
    by gzingsheim
  - Projection / Proportion / Compatibility?
    by Liquidized
  - Download URLs – double slash
    by vastur
  - map soft – writer: me
    by krzysztof
  - Unicode encoding issue – ne\_10m\_lakes.dbf
    by filter.1

---

Supported by:

© 2009 - 2022. Natural Earth. All rights reserved.


Powered by WordPress

Staff Login »
