# Supplementary material for: Global trends in smoking-attributable rheumatoid arthritis burden: Insights from GBD 2021
Source: PLoS One. 2025 Aug 5;20(8):e0329434. doi: 10.1371/journal.pone.0329434 (PMC12324138; doi:10.1371/journal.pone.0329434)
Supplement: Attachment — Submitted filename: Response to Reviewers.docx [file pone.0329434.s003.docx]

PONE-D-25-01779

Global Trends in Smoking-Attributable Rheumatoid Arthritis Burden: Insights from GBD 2021

PLOS ONE

Dear Dr. Zhou,

Thank you for submitting your manuscript to PLOS ONE. After careful consideration, we feel that it has merit but does not fully meet PLOS ONE’s publication criteria as it currently stands. Therefore, we invite you to submit a revised version of the manuscript that addresses the points raised during the review process.

Please submit your revised manuscript by Jun 22 2025 11:59PM. If you will need more time than this to complete your revisions, please reply to this message or contact the journal office at [plosone@plos.org](mailto:plosone@plos.org). When you're ready to submit your revision, log on to https://www.editorialmanager.com/pone/ and select the 'Submissions Needing Revision' folder to locate your manuscript file.

Please include the following items when submitting your revised manuscript:

- A rebuttal letter that responds to each point raised by the academic editor and reviewer(s). You should upload this letter as a separate file labeled 'Response to Reviewers'.
- A marked-up copy of your manuscript that highlights changes made to the original version. You should upload this as a separate file labeled 'Revised Manuscript with Track Changes'.
- An unmarked version of your revised paper without tracked changes. You should upload this as a separate file labeled 'Manuscript'.

If you would like to make changes to your financial disclosure, please include your updated statement in your cover letter. Guidelines for resubmitting your figure files are available below the reviewer comments at the end of this letter.

We look forward to receiving your revised manuscript.

Kind regards,

Paul Obeng, MEd, MSc., M.Phil.
Academic Editor
PLOS ONE

**Journal Requirements:**

When submitting your revision, we need you to address these additional requirements.

**1.** Please ensure that your manuscript meets PLOS ONE's style requirements, including those for file naming. The PLOS ONE style templates can be found at

<https://journals.plos.org/plosone/s/file?id=wjVg/PLOSOne_formatting_sample_main_body.pdf>

and

[https://journals.plos.org/plosone/s/file?id=ba62/PLOSOne_formatting_sample_title_authors_affiliations.pdf](https://track.editorialmanager.com/CL0/https:%2F%2Fjournals.plos.org%2Fplosone%2Fs%2Ffile%3Fid=ba62%2FPLOSOne_formatting_sample_title_authors_affiliations.pdf/1/010f0196af249fab-5dea6cd2-15d8-4270-a010-c016c23a83fc-000000/Wxa5ITdik2DKMtIxcL1Bdxnn7SETa7KtkjPW30TkWRE=210).

**Response:**

Thank you for your kind reminder. In the revised manuscript submitted, we have carefully made adjustments and modifications to the formatting according to the requirements outlined in the two provided style template documents, to ensure compliance with PLOS ONE's standard formatting requirements.

**2.** We note that your Data Availability Statement is currently as follows: [All relevant data are within the manuscript and its Supporting Information files]

Please confirm at this time whether or not your submission contains all raw data required to replicate the results of your study. Authors must share the “minimal data set” for their submission. PLOS defines the minimal data set to consist of the data required to replicate all study findings reported in the article, as well as related metadata and methods (https://journals.plos.org/plosone/s/data-availability#loc-minimal-data-set-definition).

For example, authors should submit the following data:

- The values behind the means, standard deviations and other measures reported;

- The values used to build graphs;

- The points extracted from images for analysis.

Authors do not need to submit their entire data set if only a portion of the data was used in the reported study.

If your submission does not contain these data, please either upload them as Supporting Information files or deposit them to a stable, public repository and provide us with the relevant URLs, DOIs, or accession numbers. For a list of recommended repositories, please see https://journals.plos.org/plosone/s/recommended-repositories.

If there are ethical or legal restrictions on sharing a de-identified data set, please explain them in detail (e.g., data contain potentially sensitive information, data are owned by a third-party organization, etc.) and who has imposed them (e.g., an ethics committee). Please also provide contact information for a data access committee, ethics committee, or other institutional body to which data requests may be sent. If data are owned by a third party, please indicate how others may request data access.

**Response:**

Thank you for the editor's kind reminder. To comply with PLOS ONE's data availability policy, we have prepared the minimal data set as follows: For each figure in our manuscript, we have created a dedicated folder containing all the underlying data points collated from the GBD 2021 and WHO website sources that were used to construct that figure. This packaged data has been uploaded to the submission system as Supporting Information.

**3.** We note that the grant information you provided in the ‘Funding Information’ and ‘Financial Disclosure’ sections do not match.

When you resubmit, please ensure that you provide the correct grant numbers for the awards you received for your study in the ‘Funding Information’ section.

**Response:**

Thank you for your reminder regarding the funding information. We have noted the discrepancy. The correct and consistent funding statement, which is accurately presented in our manuscript's "Funding" section and which we request be updated in the "Financial Disclosure" section of the online submission form, is as follows:

This work was supported by Scientific Research Fund of Hunan Provincial Education Department(grant number 23C0427, 23A0643), Hunan Provincial Natural Science Foundation of China (No.2021JJ40841), Research on education and teaching reform of Central South University (No. 2023jy087-3).

**4.** PLOS requires an ORCID iD for the corresponding author in Editorial Manager on papers submitted after December 6th, 2016. Please ensure that you have an ORCID iD and that it is validated in Editorial Manager. To do this, go to ‘Update my Information’ (in the upper left-hand corner of the main menu), and click on the Fetch/Validate link next to the ORCID field. This will take you to the ORCID site and allow you to create a new iD or authenticate a pre-existing iD in Editorial Manager.

**Response:**

Thank you for your kind reminder. We have now updated the corresponding author's ORCID iD in the Editorial Manager submission system as requested.

**5.** Thank you for stating the following in the Acknowledgments Section of your manuscript:

“This work was supported by Scientific Research Fund of Hunan Provincial Education Department(grant number 23C0427, 23A0643), Hunan Provincial Natural Science Foundation of China (No.2021JJ40841), Research on education and teaching reform of Central South University (No. 2023jy087-3).”

We note that you have provided additional information within the Acknowledgements Section that is not currently declared in your Funding Statement. Please note that funding information should not appear in the Acknowledgments section or other areas of your manuscript. We will only publish funding information present in the Funding Statement section of the online submission form.

Please remove any funding-related text from the manuscript and let us know how you would like to update your Funding Statement. Currently, your Funding Statement reads as follows:

 “The author(s) received no specific funding for this work.”

Please include your amended statements within your cover letter; we will change the online submission form on your behalf.

**Response:**

Thank you for your kind reminder. We have updated the Funding Statement in the journal's online submission system. The updated funding information is as follows:

This work was supported by Scientific Research Fund of Hunan Provincial Education Department(grant number 23C0427, 23A0643), Hunan Provincial Natural Science Foundation of China (No.2021JJ40841), Research on education and teaching reform of Central South University (No. 2023jy087-3).

**6.** We note that Figure 1 in your submission contain [map/satellite] images which may be copyrighted. All PLOS content is published under the Creative Commons Attribution License (CC BY 4.0), which means that the manuscript, images, and Supporting Information files will be freely available online, and any third party is permitted to access, download, copy, distribute, and use these materials in any way, even commercially, with proper attribution. For these reasons, we cannot publish previously copyrighted maps or satellite images created using proprietary data, such as Google software (Google Maps, Street View, and Earth). For more information, see our copyright guidelines: http://journals.plos.org/plosone/s/licenses-and-copyright.

We require you to either (1) present written permission from the copyright holder to publish these figures specifically under the CC BY 4.0 license, or (2) remove the figures from your submission:

**a.** You may seek permission from the original copyright holder of Figure 1 to publish the content specifically under the CC BY 4.0 license.

We recommend that you contact the original copyright holder with the Content Permission Form (http://journals.plos.org/plosone/s/file?id=7c09/content-permission-form.pdf) and the following text:

“I request permission for the open-access journal PLOS ONE to publish XXX under the Creative Commons Attribution License (CCAL) CC BY 4.0 (http://creativecommons.org/licenses/by/4.0/). Please be aware that this license allows unrestricted use and distribution, even commercially, by third parties. Please reply and provide explicit written permission to publish XXX under a CC BY license and complete the attached form.”

Please upload the completed Content Permission Form or other proof of granted permissions as an "Other" file with your submission.

 In the figure caption of the copyrighted figure, please include the following text: “Reprinted from [ref] under a CC BY license, with permission from [name of publisher], original copyright [original copyright year].”

**b.** If you are unable to obtain permission from the original copyright holder to publish these figures under the CC BY 4.0 license or if the copyright holder’s requirements are incompatible with the CC BY 4.0 license, please either i) remove the figure or ii) supply a replacement figure that complies with the CC BY 4.0 license. Please check copyright information on all replacement figures and update the figure caption with source information. If applicable, please specify in the figure caption text when a figure is similar but not identical to the original image and is therefore for illustrative purposes only.

The following resources for replacing copyrighted map figures may be helpful:

USGS National Map Viewer (public domain): http://viewer.nationalmap.gov/viewer/

The Gateway to Astronaut Photography of Earth (public domain): http://eol.jsc.nasa.gov/sseop/clickmap/

Maps at the CIA (public domain): https://www.cia.gov/library/publications/the-world-factbook/index.html and https://www.cia.gov/library/publications/cia-maps-publications/index.html

NASA Earth Observatory (public domain): http://earthobservatory.nasa.gov/

Landsat: http://landsat.visibleearth.nasa.gov/

USGS EROS (Earth Resources Observatory and Science (EROS) Center) (public domain): http://eros.usgs.gov/#

Natural Earth (public domain): <http://www.naturalearthdata.com/>

**Response:**

Thank you very much for your kind reminder. We paid close attention to copyright issues for all figures and potential conflicts of interest during our study design.

Regarding the concern about the copyright of Figure 1, we would like to clarify that the base map for this figure was generated using country boundary data (shapefile: ne_110m_admin_0_countries.shp) sourced from **Natural Earth (naturalearthdata.com)**. Natural Earth data is in the **public domain**, which means it is free for all uses, including publication under a CC BY 4.0 license. The data for tobacco smoking rates, tobacco use rates, deaths, DALYs, YLDs, and YLLs, which are plotted onto this base map, were derived from our analysis of GBD 2021 and WHO data as described in the manuscript. Therefore, Figure 1 fully complies with the PLOS ONE CC BY 4.0 licensing requirements.

Furthermore, we have added a supplementary note to the caption of Figure 1:
Figure 1. Burden of RA attributable to smoking in 2021 across 204 countries and territories. **(Base map data sourced from Natural Earth - public domain.)**

**7.** Please review your reference list to ensure that it is complete and correct. If you have cited papers that have been retracted, please include the rationale for doing so in the manuscript text, or remove these references and replace them with relevant current references. Any changes to the reference list should be mentioned in the rebuttal letter that accompanies your revised manuscript. If you need to cite a retracted article, indicate the article’s retracted status in the References list and also include a citation and full reference for the retraction notice.

**Response:**

Thank you for the reminder to carefully review our reference list. We have conducted a thorough review of all cited references in the manuscript, including those newly added during this revision process, to ensure their completeness, accuracy, and current publication status.

We confirm that, to the best of our knowledge and based on our checks, none of the references cited in our manuscript have been retracted.

**Additional Editor Comments:**

**Introduction**

**1.** While the authors mention that RA prevalence is rising globally, quantitative data or regional variability would enhance context. Consider including global or regional prevalence/incidence rates or burden metrics (e.g., DALYs or mortality). For example, in 2019, RA affected approximately X million people globally, contributing to Y DALYs and Z deaths (cite GBD source)."

**Response:**

Thank you for this valuable suggestion. We agree that providing quantitative data on the global burden of RA would significantly enhance the context of our introduction. We have revised the introduction to include specific global prevalence figures and the increase in its age-standardized prevalence rate for RA from the Global Burden of Disease (GBD) 2021 study, which is already cited as reference [3] in our manuscript.

**The original sentence was:**

Its prevalence is on the rise globally [3].

**The revised sentence, now located on page 3, lines 36-38:**

Globally, the prevalence of RA is on the rise, with an estimated 17.6 million people affected in 2020, and its age-standardized prevalence rate increasing by 14.1% since 1990 [3].

We believe this addition directly addresses your concern by providing concrete figures on the RA burden and its increasing trend, thus strengthening the rationale for our study on smoking-attributable RA.

**2.** Insufficient explanation of smoking’s pathophysiological role. Although smoking is cited as a major external risk factor, the introduction could briefly explain the mechanism by which smoking contributes to RA pathogenesis to help justify its inclusion. Add one or two sentences on how smoking induces citrullination, promotes autoantibody production, or triggers immune dysregulation relevant to RA.

**Response:**

Thank you for this insightful comment. We agree that a brief explanation of the pathophysiological link between smoking and RA would strengthen our introduction. We have now added a sentence to elaborate on this mechanism, supported by our existing reference [9] (Klareskog et al., 2011), as suggested.

**The revised text, now located on page 3, lines 42-45:**

Among these, smoking has been consistently identified as a significant external risk factor and is closely linked to the pathogenesis of RA [6-9]. Specifically, smoking is thought to promote RA development by inducing citrullination of proteins, particularly in the lungs of genetically susceptible individuals, which can lead to the generation of anti-citrullinated protein antibodies (ACPAs), key autoantibodies in RA [9].

**3.** Need to define the research gap more explicitly. The introduction could better articulate what specific gaps this study addresses. For example, is there a lack of global time-trend analyses? Have previous studies not linked smoking and RA in the context of FCTC implementation?

**Response:**

Thank you for pointing out the need to more explicitly define the research gap. We agree that this would strengthen the rationale for our study. We have revised the introduction to better articulate the specific gaps our study aims to address, particularly concerning the lack of comprehensive global time-trend analyses of smoking-attributable RA burden since the implementation of the WHO FCTC.

**We have added the following sentences before stating our study's aim, now located on page 3, lines 54-59:**

However, while the impact of smoking on RA is well-documented and the FCTC represents a global commitment to tobacco control, there is a paucity of comprehensive global analyses examining the long-term trends in smoking-attributable RA burden specifically since the FCTC's implementation. Understanding these trends is crucial for evaluating the real-world impact of tobacco control efforts on specific NCDs like RA and for guiding future public health strategies.

**4.** The final paragraph outlines the aim, but the sentence is lengthy and could be more concise and focused. For example, the objectives could be modified to “This study aims to assess global trends in the RA burden attributable to smoking since the implementation of the WHO FCTC, using data from GBD 2021 and WHO tobacco prevalence reports. Findings will inform health policy and tobacco control strategies aimed at reducing the RA burden.”

**Response:**

Thank you for this suggestion. We agree that our original statement of aims could be more concise and focused. We have revised the final paragraph of the introduction according to your an insightful suggestion.

**The original sentence was:**

This study aims to analyze and evaluate the epidemiological trends of RA burden at the global level since the implementation of the FCTC by utilizing RA burden data from the Global Burden of Disease Study 2021 (GBD 2021) and global tobacco prevalence data from the WHO. By examining the association between smoking and RA burden, the findings aim to provide evidence for developing health policies, optimizing resource allocation, and mitigating the economic and public health impacts of RA.

**The revised sentence, now located on page 3, lines 60-63:**

This study aims to assess global trends in the RA burden attributable to smoking since the implementation of the WHO FCTC, using data from GBD 2021 and WHO tobacco prevalence reports. Findings will inform health policy and tobacco control strategies aimed at reducing the RA burden.

**5.** "Interestingly, 40–70% of RA risks are attributed to non-genetic factors..." Consider removing “Interestingly,” as it adds unnecessary subjectivity.

**Response:**

We appreciate this comment. We have removed "Interestingly," from the sentence to maintain a more objective tone, as suggested.

**The revised sentence, now located on page 3, line 38, now reads:**

"40–70% of RA risks are attributed to non-genetic factors, which play an even greater role than genetic predisposition [4], highlighting the importance of studying the role of non-genetic factors in RA’s prevalence trends [5]."

**6.** Consider specifying whether the study focuses on incidence, prevalence, mortality, or DALYs for RA to avoid ambiguity.

**Response:**

Thank you for this suggestion to enhance clarity. Our study assesses the "RA burden attributable to smoking," which encompasses several key GBD metrics. While we have kept the statement of aims in the introduction concise (as per your helpful suggestion for comment 4), we have ensured that the **Methods section clearly specifies all the burden indicators analyzed.**

Additionally, these specific indicators (Deaths, DALYs, YLDs, YLLs) are consistently presented throughout our Results section and are also explicitly mentioned in the Methods subsection of our Abstract.

We believe this approach clearly defines the scope of our burden assessment within the Methods and Abstract, ensuring full transparency for the reader, while maintaining a focused and concise aim statement in the Introduction.

**Method**

**1.** While the sources of data are cited, there is insufficient detail on:

**a.**Which specific variables were extracted from GBD 2021 and WHO datasets.

**b.** How countries or regions were selected, especially with reference to completeness of data over time.

**c.** Handling of missing or inconsistent data, if any. Include a description of inclusion/exclusion criteria, any data cleaning steps, and how missing data were addressed, if applicable.

**Response:**

Thank you for these important points requesting further methodological detail. We have revised Section 2.1 ("Data Sources and Definitions") of our manuscript to provide more specific information on these aspects. We address each sub-point below:

**1a. Specific variables extracted:**

We have now clearly specified that from the GBD 2021 database, for rheumatoid arthritis (RA) attributable to smoking, we extracted both the **absolute numbers** and the **age-standardized rates (ASR, per 100,000 population)** for the following burden metrics: Deaths, Disability-Adjusted Life Years (DALYs), Years Lived with Disability (YLDs), and Years of Life Lost (YLLs). These data were obtained stratified by age group, sex, location (country/territory and GBD super-region), and year (1990-2021). Socio-Demographic Index (SDI) values for each location and year were also extracted from GBD 2021.

For tobacco exposure trends, we extracted regional and country-level data on the **age-standardized prevalence of current tobacco smoking (%)** and the **age-standardized prevalence of current tobacco use (%)**, typically for persons aged 15 years and older where specified by WHO, for selected years between 2000 and 2021 from the WHO Global Health Observatory data repository.

These clarifications have been made in Section 2.1, page 4, lines 88-89.

**1b. Country/region selection and data completeness:**

We have explicitly stated in Section 2.1 that our study aimed to include **all 204 countries and territories and 21 GBD super-regions for which GBD 2021 provides estimates from 1990 to 2021.** No additional country or region selection criteria were applied by our study beyond the availability in the GBD 2021 and WHO datasets. We acknowledge that the GBD study employs standardized methodologies, including data synthesis and statistical modeling, to generate comprehensive and comparable estimates across locations and over time, addressing potential data gaps in primary sources. Similarly, WHO provides the most complete estimates available for tobacco prevalence for the reported years.

This is now stated in Section 2.1, page 4, lines 87-97.

**1c. Handling of missing or inconsistent data:**

We have added Section 2.2 ("Data Integrity and Visualization Mapping") to clarify that we used pre-processed GBD and WHO estimates without further cleaning. For map visualizations, we applied a country name mapping strategy to improve data-shapefile merging. "No Data" regions on maps indicate actual data unavailability in our sources for that indicator or limitations of the shapefile's geographical coverage.

(Manuscript change: See Section 2.2, page 4, lines 100-108.)

**2.** The selection of years for WHO tobacco prevalence data (2000, 2005, 2007, etc.) appears arbitrary. Clarify the rationale behind choosing these years and explain whether they align with WHO data availability, FCTC milestones, or analytical objectives.

**Response:**

Thank you for this query. We apologize if our previous description made the selection of years for WHO tobacco prevalence data appear arbitrary. We have revised Section 2.1 ("Data Sources and Definitions") to provide a clearer explanation for the choice of these years.

The years for which WHO tobacco prevalence data were extracted (specifically, 2000, 2005, 2007, 2010, 2015, 2018, 2020, and 2021, as used in our analysis of trends up to 2021) were primarily dictated by the availability of comprehensive global tobacco surveillance reports and estimated data releases from the WHO for these specific time points. Our study utilized all such available reported years from WHO that fell within our overall study period (1990-2021, with WHO data starting from 2000) and aligned with our objective of analyzing trends up to 2021 (to match the GBD 2021 end year).

While the GBD 2021 data for RA burden provide continuous annual estimates, WHO typically releases comprehensive global tobacco prevalence estimates for specific sentinel years rather than annually. The selected years, based on this availability, include a pre-FCTC (Framework Convention on Tobacco Control) baseline (2000) and several post-FCTC implementation years (e.g., 2005, 2010, 2015, 2020, 2021), which is pertinent to one of our study's contextual anchors. This allows for an examination of broad trends in tobacco exposure over the past two decades, including periods before and after the FCTC (launched in 2003, entered into force in 2005) became a significant global health treaty. The figures presenting these trends (e.g., Figure 2) display the WHO data for these discrete available years.

We trust this explanation clarifies that the selection of years for WHO data was based on data availability from the source and aligns with our analytical objectives.

**3.** The linear regression model used for EAPC assumes a log-linear trend across time, but this assumption is not discussed or evaluated. A brief note on whether assumptions of linearity, homoscedasticity, and normality of residuals were checked would strengthen the rigor of the statistical analysis.

**Response:**

Thank you for this valuable comment regarding the assumptions of the linear regression model used for EAPC calculation. We acknowledge that the EAPC method, by fitting a regression model to the natural logarithm of the age-standardized rates (ASRs) against time, inherently assumes an approximately log-linear trend.

We have added a brief note in Section 2.3 (formerly 2.2, "ASR, EAPC and Percentage Change") to address this. While a formal diagnostic statistical test for all assumptions (linearity of the log-transformed rates, homoscedasticity, and normality of residuals) for every single EAPC calculated was not performed due to the extensive number of trends analyzed across multiple stratifications (regions, sexes, SDIs, etc.), we did visually inspect the scatter plots of the natural logarithm of ASRs against calendar years for major global and regional trends to qualitatively assess the appropriateness of a linear fit for summarizing the overall direction and average magnitude of change over the 32-year period (1990-2021). In general, for the long-term trends analyzed, the log-linear model appeared to provide a reasonable approximation for calculating the EAPC as a summary measure of the average annual rate of change.

It is also important to note that EAPC is a widely accepted and standard metric in epidemiological studies, including many GBD publications, for describing and comparing average trends in rates over time, even when minor deviations from perfect log-linearity might exist over shorter sub-periods. Our primary use of EAPC is as a descriptive tool to summarize the overall secular trend.

(Manuscript change: A note discussing the log-linear assumption and our qualitative assessment has been added to Section 2.3, page 4, lines 111-116.)

We believe this addition provides appropriate context regarding the use of EAPC and its underlying assumptions in our study.

**4.** No Mention of Stratification Beyond SDI. Though SDI is discussed, there's no clarity on whether analyses were stratified by sex, age group, or region, which would be important for understanding burden disparities. Explicitly state whether subgroup analyses were conducted, and if so, how these were handled statistically.

**Response:**

Thank you for pointing out this omission. We agree that explicitly stating all stratifications used in our analysis is crucial for clarity and for highlighting our examination of burden disparities. Our study indeed conducted stratified analyses by sex, age group, and GBD super-region, in addition to analyzing trends by SDI.

We have revised the Methods section to clearly state these stratifications. Specifically, in Section 2.1 ("Data Sources and Definitions"), we now detail that the extracted GBD data were stratified by these factors. Furthermore, we have added a sentence at the beginning of Section 2.3 (formerly 2.2, "ASR, EAPC and Percentage Change") to explicitly state that all trend analyses (ASR, EAPC, percentage changes) were performed and presented for these subgroups. The statistical methods for calculating ASR, EAPC, and percentage change, as described in Section 2.3, were applied consistently across all strata.

Our Results section and figures already present these stratified findings. For instance:

① Regional stratification is detailed in Section 3.1 and visualized in Figure 1 (showing country-level data which can be aggregated to regions) and Figure 2 (showing trends by GBD super-region).

② Sex and age stratification is detailed in Section 3.3 and visualized in Figure 3 (showing burden by sex and detailed age groups) and Figure 4 (showing trends by sex across GBD super-regions).

③ SDI stratification is detailed in Section 3.4 and visualized in Figure 5.

(Manuscript change: Clarification of data extraction stratification has been enhanced in Section 2.1. A new statement explicitly detailing that analyses were conducted stratified by GBD super-region, sex, and age group has been added at the beginning of Section 2.3.)

We believe these revisions, along with the clear presentation of stratified results in our manuscript, now comprehensively outline the stratified analyses undertaken in our study to explore disparities in the smoking-attributable RA burden.

**5.** Lack of Ethical Considerations Statement. While secondary data analysis typically does not require ethics approval, this should be explicitly stated for transparency. Add a sentence such as “This study used publicly available, de-identified secondary data and did not require ethical approval.”

**Response:**

Thank you for this important reminder. For transparency, and as per your suggestion, we have added a brief statement regarding ethical considerations at the end of the Methods section.

(Manuscript change: A sentence has been added at the conclusion of the Methods section (before the Results section), page 6, lines 181-184, stating that the study utilized publicly available, de-identified secondary data from GBD 2021 and WHO, and therefore, formal ethical approval was not required.)

We believe this concise addition appropriately addresses the need for an explicit ethical statement.

**Results**

**1.** The term “smoking prevalence reached 40 per 100,000” seems inaccurate or misleading. Smoking prevalence is usually expressed as a percentage of the population, not per 100,000. Please revise to correct this. Similarly, DALYs and YLLs should be reported per 100,000 population, not as raw numbers or ambiguous “reached X per 100,000” without clear context.

**Response:**

Thank you for this critical feedback. We sincerely apologize for the inaccuracies in reporting units and the lack of clarity in our initial submission. We have thoroughly revised the manuscript to address these issues comprehensively.

① **Regarding tobacco smoking rate and tobacco use rate (formerly referred to with "prevalence" in some contexts)**:

You are absolutely correct that these should be expressed as percentages (%). We have conducted a thorough review and revision of our manuscript. All textual references to "**tobacco smoking rate**" and "**tobacco use rate**" in the Results section (e.g., Sections 3.1, 3.2) have been corrected to reflect actual percentage values (%) based on our WHO source data. Correspondingly, the legends and axis labels for Figures 1A, 1B, 2A, and 2B have been revised to display these rates as "**Tobacco smoking rate (%)**" and "**Tobacco use rate (%)**", and the visual scaling of these figures now accurately represents the data in this correct unit. For instance, a statement like "smoking prevalence/rate reached 40 per 100,000" has been rectified to, for example, "tobacco smoking rate reached 40%," using the correct numerical value from the source.

**② Regarding DALYs, YLLs, Deaths, and YLDs**: We have carefully re-examined how these GBD burden metrics are reported throughout the Results section.

When referring to rate, we now consistently and explicitly use the unit "per 100,000 population" (e.g., "the age-standardized DALYs rate was Y per 100,000 population").

When discussing absolute numbers (e.g., total deaths or DALYs), we have ensured the context clearly indicates these are counts, and we have removed any ambiguous phrasing that might have incorrectly implied a rate or used "per 100,000" inappropriately with absolute numbers.

We are confident that these comprehensive revisions have significantly improved the accuracy, clarity, and scientific rigor of our results presentation.

**2.** Break down the results more cleanly by subheading or paragraphing under the main result sections: E.g., under 3.1, consider sub-labels: “Regional Trends in Deaths,” “Patterns in DALYs,” etc. This enhances readability and helps readers locate specific data more easily.

**Response:**

Thank you for this constructive suggestion to improve the readability and organization of our Results section.

We have restructured Section 3.1 ("Trends in the Global and Regional RA Burden Attributable to Smoking from 1990 to 2021"), to better delineate the findings for different burden metrics (Deaths, DALYs, YLDs, YLLs) and for different descriptive aspects (e.g., overall trends vs. regional disparities in 2021). While we may not have used the exact sub-labels suggested (to maintain a certain flow or to group related findings), we have made a concerted effort to use clearer paragraph breaks and thematic grouping of sentences to guide the reader through the data more systematically. For example, within Section 3.1, we now more clearly separate the discussion of percentage reductions in age-standardized rates for each metric from the description of regional disparities observed in the most recent year (2021).

We believe these changes have improved the overall structure and readability of the Results section, making it easier for readers to follow our findings for specific indicators.

**3.** Ensure that all references to figures and tables are correctly numbered and labeled. “Figure 1.A” through “Figure 1.F” implies subfigures—these should be clearly shown in the actual figure. Ensure consistency between text and figures.

**Response:**

Thank you for this important reminder about the correct labeling and referencing of figures and their sub-panels. We have carefully reviewed all figure and table references throughout the manuscript to ensure they are accurate and consistent.

Regarding Figure 1, we confirm that it is a multi-panel figure comprised of subfigures labeled A through F, each depicting a different indicator of smoking-attributable RA burden in 2021. We have ensured that these sub-panel labels (A, B, C, D, E, F) are clearly and prominently displayed within the composite Figure 1 itself. When referring to these specific sub-panels in the text (e.g., in Section 3.1), we now consistently use the format "Fig 1A," "Fig 1B," etc. For other figures and tables, we have also verified their numbering and the consistency of references in the text.

We believe these checks and any necessary adjustments have ensured the correct and consistent labeling and referencing of all figures and tables in the manuscript.

**4.** You’ve done well to include 95% CIs and EAPC values in the table. However, where results are highlighted in the narrative (e.g., "a decline of -50.94%"), you might include the confidence interval briefly to strengthen the statistical transparency, especially for key figures.

**Response:**

Thank you for this valuable suggestion and for acknowledging our inclusion of 95% CIs and EAPC values in the tables. We agree that incorporating 95% CIs for key EAPC values and significant percentage changes directly into the narrative text of the Results section would enhance statistical transparency and provide readers with a better sense of the precision of these estimates.

We have reviewed the Results section, particularly where EAPCs are highlighted (e.g., in Section 3.1 when discussing regional trends), and have now included the 95% CIs for these key EAPC figures directly in the text, using the data presented in Table 1.

We believe this addition strengthens the presentation of our key findings.

**5.** “...a decline of only -7.73%” consider avoiding “only” in scientific writing unless it's part of a contrastive point being emphasized.

**Response:**

Thank you for this valuable advice on scientific writing style. We agree that the use of subjective or potentially biased terms like "only" should be avoided to maintain objectivity. We have reviewed the manuscript and removed the word "only" from the sentence in question, and we have also checked for similar instances throughout the text to ensure a neutral tone.

We appreciate your guidance on improving the objectivity of our language.

**6.** "These findings highlight..." → consider beginning a new paragraph here to separate description of data from interpretation.

**Response:**

Thank you for this suggestion to improve the separation between data description and its initial interpretation.

We have reviewed Section 3.1 where a sentence originally beginning "These findings highlight..." served to summarize the EAPC trends. Following previous revisions for clarity and conciseness, this interpretive sentence now reads "These varied regional trends likely reflect differences in the implementation and effectiveness of tobacco control policies, healthcare access and quality, and prevailing cultural practices related to smoking."

As per your current suggestion, this sentence, which summarizes the potential reasons for the observed varied regional trends in smoking-attributable RA burden from 1990 to 2021, has now been made into its own concise, separate paragraph. This paragraph immediately follows the detailed presentation of the EAPC trends for Deaths, DALYs, YLDs, and YLLs, and precedes the discussion of regional disparities in 2021, thereby providing a clearer demarcation.

We believe this adjustment, building upon earlier revisions, further enhances the clarity and logical flow of this section by distinctly separating data presentation from its immediate contextualization.

**7.** Consistently use full terms on first mention in each subsection (e.g., "Years Lived with Disability (YLDs)").

**Response:**

Thank you for this important reminder regarding the consistent use of full terms and their abbreviations, as per the editorial comments. We agree that defining abbreviations on their first use within each subsection significantly enhances clarity and readability for our readers.

We have meticulously reviewed the entire manuscript, encompassing all main sections (Abstract, Introduction, Methods, Results, Discussion) and their respective subsections. This review was to ensure that all key terms and abbreviations – such as DALYs (Disability-Adjusted Life Years), YLDs (Years Lived with Disability), YLLs (Years of Life Lost), ASR (Age-Standardized Rate), EAPC (Estimated Annual Percentage Change), SDI (Socio-Demographic Index), RA (Rheumatoid Arthritis), WHO (World Health Organization), GBD (Global Burden of Disease), and FCTC (Framework Convention on Tobacco Control) – are written out in full upon their first mention within each individual subsection. This is followed by the abbreviation in parentheses. Subsequent mentions of the term within that same subsection then utilize the abbreviation.

We believe this systematic approach to defining abbreviations, in line with your guidance, will significantly improve the overall readability and accessibility of our manuscript.

**Discussion**

**1.** While you note the increased absolute Deaths and DALYs, there could be more exploration of why the smoking-attributable burden hasn't declined as sharply as overall smoking rates—e.g., delayed effects of smoking, demographic transitions.

**Response:**

Thank you for this insightful suggestion. We agree that a more in-depth exploration of the reasons behind the slower decline in smoking-attributable RA burden compared to overall smoking rates would significantly strengthen our discussion.

We have revised the Discussion section (now the second paragraph) to specifically address this. We now elaborate on several contributing factors, including:

① The well-documented lag effect between smoking exposure and the clinical manifestation of RA, meaning current burden reflects past smoking behaviors [27].

② Global demographic transitions, such as overall population growth and, critically, population aging, which increases the proportion of individuals in higher RA-risk age groups with longer cumulative smoking exposure [28, 29].

③ Potential impacts of advancements in RA management leading to increased survival durations with the disease, thereby affecting YLDs and absolute DALYs, even if mortality rates decrease [30].

We believe this expanded discussion provides a more nuanced understanding of the complexities involved in translating tobacco control successes into immediate and proportional reductions in smoking-attributable disease burden.

**2.** You mention comorbidities in older adults—can you tie this back to clinical practice implications, such as integrated care?

**Response:**

Thank you for prompting us to elaborate on the clinical practice implications of comorbidities in older adults with smoking-attributable RA.

We have revised the paragraph discussing age-related burden (now the fifth paragraph of the Discussion). We now explicitly link the observation of high RA burden and the influence of comorbidities in older, smoking-exposed individuals to the need for integrated care approaches. We suggest that such approaches should combine RA management with proactive screening and management of smoking-related comorbidities, alongside continued smoking cessation support, to mitigate further health deterioration in this vulnerable population [35].

We believe this addition better highlights the practical clinical relevance of our findings regarding older RA patients with a history of smoking.

**3.** The assertion that low SDI countries may underestimate burden due to data gaps or economic constraints is plausible but needs a supporting reference.

**Response:**

Thank you for pointing out the need to support this assertion with a reference. We agree that this is important for substantiating the claim.

In the paragraph discussing SDI-related differences (now the seventh paragraph of the Discussion), when we state that the lower reported burdens in low SDI countries might be an underestimation, we now explicitly mention that "The availability and quality of health data, including cause-of-death registration and RA diagnosis capabilities, are often more limited in low SDI settings, and economic constraints can impede access to care and thus affect disease reporting and burden estimation." We have now added a supporting reference [37] for this statement, in addition to our existing reference [36] (which was [32] in the previous draft).

We believe this revision, with the added citation, strengthens the plausibility of our assertion regarding potential underestimation of burden in low SDI countries.

**4.** “...all indicators (Deaths, DALYs, YLDs, and YLLs) , with...” → remove the space before the comma.

**Response:**

Thank you for catching this typographical error. We have carefully reviewed the manuscript and removed the erroneous space before the comma in the specified instance, and have also performed a general check for similar punctuation errors throughout the document to ensure correct formatting.

**Reviewers' comments:**

**Comments to the Author**
**2. Has the statistical analysis been performed appropriately and rigorously?**

Reviewer #1: Yes

Reviewer #2: I Don't Know

**3. Have the authors made all data underlying the findings in their manuscript fully available?**

The [PLOS Data policy](http://track.editorialmanager.com/CL0/http:%2F%2Fwww.plosone.org%2Fstatic%2Fpolicies.action%23sharing/1/010f0196af249fab-5dea6cd2-15d8-4270-a010-c016c23a83fc-000000/bPZEV8zd6yAFP9Bo86wXKgacGUVwmhaxRonW2fEvX-o=210) requires authors to make all data underlying the findings described in their manuscript fully available without restriction, with rare exception (please refer to the Data Availability Statement in the manuscript PDF file). The data should be provided as part of the manuscript or its supporting information, or deposited to a public repository. For example, in addition to summary statistics, the data points behind means, medians and variance measures should be available. If there are restrictions on publicly sharing data—e.g. participant privacy or use of data from a third party—those must be specified.

Reviewer #1: Yes

Reviewer #2: Yes

**4. Is the manuscript presented in an intelligible fashion and written in standard English?**

PLOS ONE does not copyedit accepted manuscripts, so the language in submitted articles must be clear, correct, and unambiguous. Any typographical or grammatical errors should be corrected at revision, so please note any specific errors here.

Reviewer #1: Yes

Reviewer #2: Yes

**5. Review Comments to the Author**

Please use the space provided to explain your answers to the questions above. You may also include additional comments for the author, including concerns about dual publication, research ethics, or publication ethics. (Please upload your review as an attachment if it exceeds 20,000 characters)

**Reviewer #1:**

**1.** The manuscript presents an extensive examination of the relationship between smoking and the burden of rheumatoid arthritis (RA) across a wide range of populations and regions. Overall, the study is well-conducted and offers valuable insights using up-to-date data sources, such as the GBD 2021 and WHO databases. However, several improvements are necessary to further enhance clarity and presentation before publication.

**Response:**

We sincerely thank the reviewer for their positive assessment of our study's scope, conduct, and the value of its insights based on up-to-date GBD 2021 and WHO data. We also appreciate the constructive feedback highlighting areas where clarity and presentation can be further enhanced. We have carefully considered all specific comments provided by the reviewer (and the editor) and have made corresponding revisions throughout the manuscript to address these points, aiming to improve the overall quality and readability of our paper. We detail these specific changes in our responses to the subsequent points.

**2.** Firstly, while the epidemiological metrics (e.g., Disability-Adjusted Life Years [DALYs], Years of Life Lost [YLLs], Years Lived with Disability [YLDs], Socio-Demographic Index [SDI], Uncertainty Interval [UI], Estimated Annual Percentage Change [EAPC], and Age-Standardized Rate [ASR]) are central to the analysis, their full forms should be clearly defined at first use. This change would ensure that readers from various disciplines can follow the arguments without confusion.

**Response:**

Thank you for highlighting the importance of clearly defining all epidemiological metrics and abbreviations at their first use. We agree that this is crucial for ensuring the manuscript is accessible and understandable to a broad multidisciplinary audience.

In response to this comment, and also in line with previous editorial feedback, we have conducted a meticulous review of the entire manuscript (Abstract, Introduction, Methods, Results, and Discussion sections, including their subsections). We have ensured that all key epidemiological metrics and other abbreviations mentioned – specifically:

Disability-Adjusted Life Years (DALYs),

Years of Life Lost (YLLs),

Years Lived with Disability (YLDs),

Socio-Demographic Index (SDI),

Uncertainty Interval (UI),

Estimated Annual Percentage Change (EAPC),

Age-Standardized Rate (ASR),

Rheumatoid Arthritis (RA),

World Health Organization (WHO),

Global Burden of Disease (GBD), and

Framework Convention on Tobacco Control (FCTC) – are written out in full upon their first mention within each major section (and principal subsections where appropriate for clarity, particularly in the Methods and Results sections), followed by the abbreviation in parentheses. Subsequent mentions within that same section/subsection then use the abbreviation.

We are confident that this systematic approach to defining terms will significantly improve the clarity and accessibility of our paper.

**3.** Secondly, the presentation of mathematical equations within the Methods section is currently embedded in the text and could be reformatted into stand-alone display equations with proper numbering and consistent variable notation. Enhanced clarity in the display of equations will aid in understanding the statistical approaches used and in verifying the methods.

**Response:**

Thank you for your comment regarding the presentation of mathematical equations. We would like to clarify that in our submitted manuscript (and the current revised version), the key mathematical equations in the Methods section (for ASR, the EAPC regression model, EAPC calculation, and DALYs calculation, now Equations 1-4 in Sections 2.3 and 2.4) were indeed formatted as stand-alone display equations, each with sequential numbering.

To illustrate this, please see the screenshot below showing Equations 1-4 as they appear in our revised manuscript:

We apologize if there was an issue with how these equations were rendered or displayed in the version you reviewed, which might have made them appear embedded or unclear. We have double-checked the formatting in our source document to ensure they are properly set as distinct, numbered display equations using standard equation editors.

We hope this clarification and the provided visual evidence address your concerns, and we are confident that the equations are presented in a clear and standard manner in our manuscript.

**4.** Furthermore, the manuscript would benefit from a more consistent formatting of tables and figures. Table headers and figure captions should uniformly report units (e.g., per 100,000 population) and be detailed enough to stand alone. Special attention should be given to ensuring that all figures are of high resolution and that legends are clearly labeled.

**Response:**

Thank you for these important recommendations for improving the consistency, clarity, and overall presentation quality of our tables and figures. We have undertaken a comprehensive review and revision of all tables and figures in accordance with your valuable feedback.

**Uniform reporting of units and enhanced detail in table headers and figure captions:**

① We have carefully revised Table 1. The main column group previously titled "Age-standardised Percentage Attributable to Smoking" has been corrected to accurately reflect that the presented data are "Age-Standardised Rate (per 100,000 population) Attributable to Smoking," with sub-headers now clearly indicating "ASR (95% UI)" for 1990 and 2021. The title of Table 1 has also been made more descriptive, and clear footnotes defining all abbreviations (ASR, UI, CI, EAPC) and units have been added to ensure it can be understood independently.

② For Figure 1, the legends for sub-panels A (Tobacco smoking rate) and B (Tobacco use rate) now correctly display the unit as percentage (%), and the data ranges in the legend reflect these percentage values. For sub-panels C-F (Deaths, DALYs, YLDs, YLLs rates), the unit "per 100,000 population" is consistently used throughout their legends and captions.

③ For Figure 2, the Y-axis labels for sub-panels A (Tobacco smoking rate) and B (Tobacco use rate) have been corrected to "Tobacco smoking rate (%)" and "Tobacco use rate (%)" respectively, with the Y-axis scales representing actual percentage values. The Y-axis labels for sub-panels C-F consistently use "per 100,000 population".

④ We have ensured that all figures (Figures 1-5) submitted with the revised manuscript are of high resolution (saved at 300 DPI) to meet publication quality standards.

We are confident that these comprehensive revisions to our tables and figures have substantially improved their consistency, clarity, and overall presentation quality, making them more accessible and informative for the reader.

**5.** Additionally, while the authors provide a detailed account of their methodological approach, incorporating a brief discussion of data quality—especially in low-income regions—and sensitivity analyses would strengthen the overall robustness of the study. Finally, minor language and typographical errors need correction, and administrative text should be streamlined to maintain focus on the science.

**Response:**

Thank you for these insightful suggestions to further strengthen our study's robustness and presentation. We address each component below:

**① Discussion of data quality (especially in low-income regions):**

We acknowledge the importance of discussing data quality, particularly for estimates from low-income regions. In our revised Discussion section (specifically in the paragraph addressing SDI-related differences, and also in the Limitations subsection), we have expanded on this. We now more explicitly state that the availability and quality of health data, including cause-of-death registration and RA diagnosis capabilities, can be more limited in low-SDI settings, which might lead to underestimation or wider uncertainty in the GBD estimates for these regions, even with the sophisticated modeling techniques employed by the GBD collaboration. (Page 17, lines 430-437 and lines 439-442)

**② Sensitivity analyses:**

While a full-scale sensitivity analysis by, for example, systematically varying input parameters or using alternative data sources for all 204 countries over 32 years was beyond the scope of this descriptive epidemiological study based on GBD estimates, we acknowledge its potential value. The GBD study itself incorporates extensive uncertainty analysis, providing 95% Uncertainty Intervals (UIs) for all its estimates, which we have reported throughout our manuscript (e.g., in Table 1 and for key figures in the Abstract and Discussion). These UIs inherently reflect the uncertainty arising from various sources, including data sparseness and model specifications. We have ensured that our interpretation of findings considers these UIs. We have also added a sentence to our Limitations section acknowledging that the absence of further study-specific sensitivity analyses (beyond those inherent in GBD's UIs) could be a limitation. (Page 18, lines 447-453)

**③ Language, typographical errors, and administrative text:**

We have thoroughly proofread the entire manuscript to correct minor language and typographical errors. We have also reviewed the text to streamline any administrative or non-essential phrasing to ensure a clear focus on the scientific content.

**We appreciate your comprehensive feedback, which we believe has helped us to improve the rigor and clarity of our manuscript.**

**Reviewer #2:**

This manuscript provides a comprehensive picture of how smoking relates to rheumatoid arthritis outcomes worldwide. There were a few points of inconsistency throughout the manuscript, it is unclear if there may have been data missing that impacted the findings, and the methods could use more clarity.

**1.** Financial disclosure section says that “The author(s) received no specific funding for this work.” but there is a funding section of the paper which states “This work was supported by Scientific Research Fund of Hunan Provincial Education.” Please edit for accuracy and consistency.

**Response:**

Thank you for pointing out this critical inconsistency between the Financial Disclosure statement entered into the submission system and the Funding section detailed within our manuscript. We sincerely apologize for this error during the initial submission process.

We will rectify this discrepancy during the resubmission of our revised manuscript by updating the Financial Disclosure information within the journal's submission system to precisely match the funding details provided in our paper. This will ensure complete accuracy and consistency between the manuscript and the submission metadata.

**2.** Similarly, the data availability statement in entry fields at the start of the submission says, “Yes - all data are fully available without restriction” instead of including the data availability statement in the paper which includes the location of the data (lines 356-358, “The datasets analysed during the current study are available in the [Global Burden of”).

**Response:**

Thank you for identifying this discrepancy between the data availability information provided in the submission system's entry fields and the detailed Data Availability Statement within our manuscript. We apologize for this inconsistency.

You are correct that our manuscript (lines 356-358 in the original submission, now page 19, lines 479-481 in the revised manuscript, under the "Availability of data and materials" section) provides the specific location of the datasets: "The datasets analysed during the current study are available in the [Global Burden of Disease Study] repository, [https://ghdx.healthdata.org/gbd-2021/sources]." This detailed statement accurately reflects how to access the GBD data. The WHO data for tobacco prevalence are also publicly available from the WHO Global Health Observatory, as cited [18].

We appreciate you bringing this to our attention to ensure full consistency and clarity regarding data availability.

**3.** Abstract: Why is smoking referred to as an “external” risk factor? In the intro it is clear when you refer to genetic risk factors and environmental risk factors, but the use of “external” seems undefined.

**Response:**

Thank you for pointing out the potentially undefined use of the term "external" risk factor in the Abstract. We agree that "environmental" is a more standard and clearer term in this context, especially as it aligns with our usage in the Introduction.

We have revised the first sentence of the Abstract's Background section. The term "external" has been replaced with "environmental" to maintain consistency with the Introduction and to use more widely understood terminology.( Page 2, line 2)

**4.** Abstract is difficult to understand due to use of abbreviations without definitions throughout --- recommend to not include undefined abbreviations in the abstract.

**Response:**

Thank you for this important feedback regarding the use of abbreviations in the Abstract. We agree that all abbreviations used in the Abstract should be defined upon their first mention to ensure it is fully understandable as a stand-alone summary.

We have carefully reviewed and revised the Abstract to address this. All key abbreviations such as RA (Rheumatoid Arthritis), GBD (Global Burden of Disease), DALYs (Disability-Adjusted Life Years), YLDs (Years Lived with Disability), YLLs (Years of Life Lost), SDI (Socio-Demographic Index), UI (Uncertainty Interval), and WHO (World Health Organization) are now defined with their full term followed by the abbreviation in parentheses at their first appearance within the Abstract. Subsequent mentions within the Abstract then use the abbreviation.

**5.** Methods, “In GBD 2021, causes are categorized into four levels, ranging from Level 1 (communicable, maternal, neonatal, and nutritional diseases) to Level (latent tuberculosis infection).” Could you add in what these are levels of and how this information is important to the analysis in this paper?

**Response:**

Thank you for this question seeking clarification on the GBD cause hierarchy and its relevance to our study. We realize that the brief mention of these levels might have been unclear without further context.

The GBD cause hierarchy is a structured classification system used by the GBD study to organize all diseases, injuries, and risk factors into progressively detailed levels for systematic analysis and reporting of health loss. Level 1 represents the broadest categories (Group I: Communicable, maternal, neonatal, and nutritional diseases; Group II: Non-communicable diseases (NCDs); Group III: Injuries). Level 2 further divides these, Level 3 provides more specific cause categories (e.g., Rheumatoid Arthritis falls under Musculoskeletal disorders, which is a Level 2 cause under NCDs), and Level 4 offers the most granular detail for some causes.

Upon reflection, we agree with the implicit point that the detailed GBD cause hierarchy levels (ranging from Level 1 to Level 4) are not directly critical to the specific analysis or understanding of our study, which focuses solely on Rheumatoid Arthritis (a Level 3 cause) and its burden attributable to smoking. Our primary intention in mentioning it was to provide general context about the GBD framework.

To improve clarity and focus, we have revised Section 2.1 ("Data Sources and Definitions") to remove the general description of the four-level GBD cause hierarchy. Instead, we now simply state that RA is classified as a specific cause within the GBD framework under musculoskeletal disorders, and provide its GBD Level 3 classification for completeness, without detailing the entire hierarchy, as this broader information does not directly impact our study's methods or results concerning smoking-attributable RA.(Page 4,lines 92-93)

**6.** Lines 172-173 need more information. They state, “In some Eastern European countries, smoking prevalence reached 40 per 100,000 while in sub-Saharan Africa, it was below 10 per 100,000.” These statistics appear incorrect, because smoking prevalence can be as high as 40% of the population, or 40 of every 100 people, in some countries. Either these statistics need to be updated or the sentence needs clarification to explain what these numbers are. Figure 1 similar shows in the keys that the statistics are per 100,000. At least for tobacco smoking and use, these numbers are actually likely per 100, not 100,000. I cannot speak to the other statistics because I am not as familiar with rheumatoid arthritis.

**Response:**

Thank you for your vigilance in pointing out this significant error in our reporting of smoking prevalence units and values in both the text (lines 172-173 of the original manuscript, now Section 3.1) and in the legend of Figure 1 (specifically Figure 1A and 1B).

You are absolutely correct; smoking prevalence and tobacco use rates are standardly reported as percentages (%) of the population, not as rates per 100,000. This was a serious oversight in our initial presentation for which we apologize.

We have undertaken a thorough correction of this issue throughout the manuscript:

① Textual Corrections: The sentence in Section 3.1 and any similar statements discussing smoking or tobacco use rates have been revised. The numerical values are now correctly presented as percentages based on our WHO source data, and the unit is explicitly stated as "%". For example, the illustrative sentence now reads: "For example, in some Eastern European countries, tobacco smoking rates reached approximately 40%, while in sub-Saharan Africa, they were below 10%." (page 7,lines 213-217)

② Figure Corrections: The legends for Figure 1A (Tobacco smoking rate) and Figure 1B (Tobacco use rate) have been completely revised. The titles now correctly indicate "rate (%)", and the data ranges in the color legend are now based on and display actual percentage values. Similarly, the Y-axis labels for Figure 2A and 2B have also been corrected to show "rate (%)" with appropriate percentage scales.

③ Data Consistency: We have re-confirmed that the underlying data for these figures and textual descriptions are the age-standardized prevalence estimates from WHO, which are provided as percentages.

We are confident that these extensive corrections have rectified the inaccuracies regarding the reporting of tobacco smoking and use rates, ensuring they are now presented in the standard and correct percentage format. We appreciate you bringing this critical point to our attention.

**7.** I’m having difficulty understanding table 1 DALYs. For example, Africa shows 6.22 for 1990 and 6.52 for 2021, but the % change is -41.78% --- this is confusing because there appears to be an increase from 1990 to 2021, not a decrease, and for example, the Americas has a similar change score below it, but the change from 10.91 in 1990 to 4.15 in 2021 appears to be a larger change and represents a decrease instead of an increase. It may be useful to define percentage change in the section titled, “2.2 ASR, EAPC and Percentage Change” --- the only information on percentage change in this section appears to be, “Additionally, this study used percentage change to represent the trend of the RA burden attributable to smoking in 2021 compared to 1990.” Other areas of Table 1 similarly show a decrease under percentage change but the 1990 to 2021 numbers appear to show an increase.
**Response:**

Thank you for your careful review of Table 1 and for pointing out the previous inconsistencies which understandably caused confusion. We sincerely apologize for these errors in our initial submission. We have undertaken a thorough revision of Table 1, including re-verification and recalculation of values, to ensure accuracy and internal consistency.

① Clarification of Table 1 Content:

The primary data presented in the first two data columns of Table 1 (for 1990 and 2021) represent the Age-standardised Percentage Attributable to Smoking (i.e., Smoking Attributable Fraction, SAF, expressed in %) for each burden metric (Deaths, DALYs, YLDs, YLLs), along with their 95% Uncertainty Intervals (UIs). The table title and column headers have been revised to clearly reflect this.

② Recalculation and Correction of "Percentage change":

The "Percentage change 1990-2021 (%)" column now accurately reflects the percentage change in these SAF values from 1990 to 2021. All values in this column have been re-calculated and verified. For the example you cited, Africa DALYs, the SAF changed from 6.22% in 1990 to 6.52% in 2021, and the "Percentage change" is now correctly reported as +4.92% (95% UI: 5.19% to 3.06% from your screenshot), indicating an increase in the attributable fraction for this specific case. (Note: The UI for Pct Change (5.19, 3.06) seems unusual as lower is greater than upper, please double check this specific UI in your source/Table 1). We have performed similar checks and corrections for all rows in Table 1.

③ Clarification of EAPC:

The "EAPC 1990-2021 (%)" column presents the Estimated Annual Percentage Change of the SAFs, calculated by fitting a log-linear regression model to the annual SAF data from 1990 to 2021. It represents the average annual rate of change of the SAF over this period. For Africa DALYs, the EAPC of SAF remains -1.84% (95% CI: -1.95% to -1.73%), indicating that while the SAF point estimate for 2021 was slightly higher than for 1990, the overall annual trend across the entire 32-year period showed an average decrease. This highlights the difference between a direct two-point percentage change and a modeled annual trend over multiple years.

④ Definition of "Percentage change":

Definition of "Percentage change": As suggested, we have ensured that Section 2.3 ("ASR, EAPC and Percentage Change") now explicitly defines how the "Percentage change" reported in Table 1 is calculated, as detailed in Equation (2) within Section 2.3.

We sincerely apologize again for the previous errors and any confusion caused. We are confident that the revised Table 1 is now accurate, internally consistent, and clearly presented, and that the methodological descriptions adequately explain all calculations.

**8.** To happen during copy editing --- Figure 1 will need a higher pixel count. Very hard to read the graph legend, even after zooming in it is blurry. This applies to other figures as well.

**Response:**

Thank you for pointing out the issue with the resolution of Figure 1 and potentially other figures in our initial submission. We apologize for the poor quality and any difficulty this caused in reading the legends and details.

We have taken this feedback very seriously and have ensured that all figures (Figures 1-5) included in the revised manuscript are now generated and saved at a high resolution (300 DPI) to ensure clarity and legibility, even when zoomed in.

Specifically for Figure 1, which is a multi-panel map, we have re-rendered it ensuring that the map details, country boundaries, color gradations, and especially the legend (including the title, color patches, data range labels, and "No Data" indicator) are crisp and clear. We have applied similar attention to detail for all other figures to improve their overall presentation quality. We will also utilize the PLOS ONE Preflight Analysis and Conversion Engine (PACE) tool before final resubmission to check figure compliance with journal standards.

We are confident that the revised figures now meet the necessary quality standards for publication and are much easier to interpret.

**9.** Recommend checking the data in Figure 1, and assigning a specific color for countries where these is no data. For example, the figure shows that Russia has low smoking and tobacco use rates; however, other sources show that Russia has very high tobacco use and smoking rates:

https://globalactiontoendsmoking.org/research/tobacco-around-the-world/russia/ .

Potentially the data are missing for some countries and “0” was erroneously assigned. If it is found that data are missing from these figures with “0” erroneously assigned, I suggest that the authors check all data and statistics in the paper. Similarly, you note on lines 326-327, “First, data from low-income countries in the GBD database may be incomplete, potentially underestimating the actual burden.” Could you provide more information on how the data maybe incomplete, why you’re unable to identify if they are or are not incomplete, and how the lack of data may have impacted the statistics you report.

**Response:**

Thank you for these very important observations and requests for clarification regarding data accuracy in Figure 1, the representation of "No Data," and the discussion of data completeness, particularly for low-income countries. We have addressed these points as follows:

**① Data Verification for Figure 1 (e.g., Russia) and "No Data" Representation:**

a. We appreciate you bringing the specific example of Russia's smoking rates to our attention and providing an alternative data source. We have re-verified the data for smoking prevalence (Figure 1A) and tobacco use prevalence (Figure 1B) for all countries, including Russia, against our WHO source data for the year 2021 (the year depicted in Figure 1). Our figures are based on the age-standardized prevalence estimates as reported by WHO for that year. Discrepancies with other sources can arise due to different methodologies, age groups covered, years of data collection, or definitions of smoking/tobacco use. We rely on the WHO's standardized global estimates for consistency in our GBD-anchored study.

b. Regarding the assignment of "0" for missing data: Our data processing and mapping pipeline does NOT assign "0" to countries with missing data. Instead, as a result of our previous revisions (prompted by other reviewer/editor comments and our own debugging of the map generation code), countries for which data are unavailable in our source files (e.g., smoking.xlsx, DALYs.xlsx) or that could not be matched to the shapefile (after applying country name mapping) are now explicitly rendered with a distinct pattern (light grey with diagonal hatches) and are labeled as "No Data" in the legend of Figure 1. We have confirmed that Russia is indeed mapped and colored based on its 2021 WHO-reported smoking/use rate in our revised Figure 1A and 1B. The color it displays corresponds to the data range its specific rate falls into. (If Russia was previously shown as "No Data" or with an incorrect color due to matching issues, this has been rectified by our country name mapping efforts.) We have thoroughly checked to ensure no "0" values were erroneously assigned in place of missing data for any country in Figure 1.

**② Elaboration on Data Incompleteness in Low-Income Countries:**

a. We have expanded our discussion on data limitations for low-income countries in both the Discussion section (paragraph on SDI-related differences) and the Limitations subsection.

b. We now provide more context on how data might be incomplete or of variable quality: This can include less comprehensive vital registration systems leading to under-ascertainment or misclassification of causes of death (affecting Deaths and YLLs estimates for RA); limited access to healthcare and diagnostic facilities leading to under-diagnosis and under-reporting of RA prevalence (affecting YLDs and DALYs estimates); and sparser primary data inputs for risk factors like smoking prevalence in some GBD modeling regions.

c. Why it's challenging to definitively identify the full extent of incompleteness from our secondary data position: As users of the GBD and WHO compiled estimates, we rely on the extensive work these organizations do to model and impute missing data and to quantify uncertainty. While GBD provides uncertainty intervals (UIs) that reflect data sparseness战争, the exact degree of underestimation beyond what is captured by UIs can be difficult for secondary analysts to precisely quantify without access to all primary data sources and GBD's internal modeling parameters. Our statement reflects the general understanding in global health that data systems in low-resource settings often face greater challenges.

d. How lack of data might impact reported statistics: We now state more clearly that this potential underestimation or wider uncertainty means that the true burden of smoking-attributable RA in some low-SDI countries could be higher than our reported point estimates, and that trends might be less certain. This underscores the need for strengthening health information systems globally.

We are grateful for the opportunity to clarify these crucial aspects of our data presentation and interpretation.

**10.** Line 231, “Among individuals aged 70-4” should likely be “70-74” ---recommend checking.
**Response:**

Thank you for catching this typographical error in the age group designation. You are correct; it should indeed be "70-74". We have corrected this in the revised manuscript.(page 13, line 301)

**11.** This paper could use a more thorough discussion of limitations and methods.

**Response:**

Thank you for this feedback. We acknowledge the importance of a robust Methods section and a comprehensive discussion of study Limitations. In response to this comment, as well as specific points raised by both reviewers and the editor, we have made several key revisions to enhance these sections:

**① Methods section enhancement:**

As detailed in our responses to Reviewer #1 (Point 3, Point 5) and Reviewer #2 (Point 1, Point 2, Point 3, Point 5), we have significantly expanded and clarified various aspects of our Methods section. This includes:

a. More precise descriptions of variables extracted from GBD and WHO (R2, P1a).

b. Clarification on country/region selection and data completeness (R2, P1b).

c. Detailed explanation of how missing data were handled for map visualization, including our country name mapping strategy (R2, P1c).

d. Rationale for the selection of WHO data years (R2, P2).

e. Discussion of the EAPC model's log-linear assumption (R2, P3).

f. Streamlining the description of the GBD cause hierarchy to focus on RA's classification (R2, P5).

g. Reformatting of mathematical equations for clarity (R1, P3).

h. Explicit statement of stratified analyses (Editor, R2, P4).

i. Addition of an ethical considerations statement (Editor, R2, P5).

We believe these cumulative revisions have made our Methods section (Section 2) substantially more detailed, transparent, and robust.

**② Limitations section enhancement (within Discussion):**

As addressed in our response to Reviewer #1 (Point 5) and Reviewer #2 (Point 9), we have expanded the Limitations subsection within our Discussion. This now includes:

a. A more detailed acknowledgment of potential data quality issues and underestimation of burden in low-income/low-SDI countries, including how these limitations might impact findings and the challenges in quantifying their full extent from a secondary analysis perspective.

b. A statement acknowledging that while GBD provides uncertainty intervals, no additional study-specific sensitivity analyses were performed.

c. We have retained our original limitations regarding the discrete nature of WHO tobacco data years and the lack of analysis on interactions between smoking characteristics and other risk factors.

We feel these additions provide a more thorough and critical appraisal of our study's potential limitations.

We hope that these substantial revisions to both the Methods and the Limitations sections address your concerns and have improved the overall rigor and comprehensiveness of our manuscript.

**We sincerely thank you once again for your many constructive comments, which have been invaluable in helping us to significantly improve the fluency and rigor of this manuscript.**

While revising your submission, please upload your figure files to the Preflight Analysis and Conversion Engine (PACE) digital diagnostic tool, [https://pacev2.apexcovantage.com/](https://track.editorialmanager.com/CL0/https:%2F%2Fpacev2.apexcovantage.com%2F/1/010f0196af249fab-5dea6cd2-15d8-4270-a010-c016c23a83fc-000000/RlXExK7tWRvO4hJxs5lkeO2Em60Qxb3uDXzTrif7tvc=210). PACE helps ensure that figures meet PLOS requirements. To use PACE, you must first register as a user. Registration is free. Then, login and navigate to the UPLOAD tab, where you will find detailed instructions on how to use the tool. If you encounter any issues or have any questions when using PACE, please email PLOS at [figures@plos.org](mailto:figures@plos.org). Please note that Supporting Information files do not need this step.
